# Artificial Intelligence, Academic Publishing, Scientific Writing, Peer Review, and Ethics

**DOI:** 10.21470/1678-9741-2023-0377

**Published:** 2024-07-15

**Authors:** Somsri Wiwanitmkit, Viroj Wiwanitkit

**Affiliations:** 1 Private Academic and Editorial Consultant, Bangkok, Thailand; 2 Center for Global Health Research, Saveetha Medical College, Saveetha Institute of Medical and Technical Sciences, Tamil Nadu, India

Dear Editor,

We follow the topic on “Artificial Intelligence is Irreversibly Bound to Academic Publishing — ChatGPT is Cleared for Scientific Writing and Peer Review^[1]^.” The convergence of artificial intelligence (AI), academic publishing, scientific writing, peer review, and ethics creates a fascinating and complex terrain. As AI technologies progress, they have the potential to transform several areas of academic publishing, including scientific writing and peer review. However, in order to ensure the responsible and ethical use of AI in these domains, ethical considerations must be properly evaluated and addressed. The legitimacy and transparency of AI-generated content in scientific writing is one ethical concern. While AI can assist in the generation of material such as abstracts, introductions, or even full manuscripts, it is critical to clearly highlight the involvement of AI systems in such content creation.

Transparency is critical for preserving the integrity of scientific writing and avoiding deceiving readers or misrepresenting the human involvement to the research process. Another ethical consideration is the possibility of data bias or manipulation by AI systems. AI algorithms are trained on existing data, and if the training datasets are biased or contain incorrect information, the outcomes will be skewed or wrong. This has far-reaching ramifications for scientific study and publication. To address this ethical challenge, it is critical to ensure that AI systems are trained on broad and unbiased datasets and to apply rigorous validation mechanisms.

The incorporation of AI has an impact on the peer review process, which is a cornerstone of academic publishing. AI can help to automate certain components of peer review, such as locating potential reviewers and doing preliminary evaluations of papers. However, it is critical to guarantee that the human component of peer review is not jeopardized. Maintaining the integrity and objectivity of the review process, avoiding conflicts of interest, and ensuring that human reviewers are recognized and respected for their competence are all ethical considerations. Furthermore, the use of AI in academic publishing raises concerns about intellectual property and plagiarism. AI systems have easy access to enormous amounts of published content, which could result in unintended or purposeful plagiarism. To ensure that AI systems are trained to respect copyright laws and appropriately attribute sources, ethical rules should be set.

Furthermore, the repercussions of AI-generated content or AI-assisted peer review should be thoroughly investigated. While AI can improve efficiency and accuracy, it may also have unforeseen consequences or restrictions. Ethical considerations should include assessing the influence of AI on the quality and credibility of published research, as well as potential biases or inequalities caused by the usage of AI systems. Collaboration between researchers, publishers, and AI developers is critical for navigating these ethical problems. To ensure openness, accountability, and justice, clear norms and best practices should be established to govern the use of AI in academic publishing. To adapt to growing issues and breakthroughs, AI systems’ performance and ethical implications must be evaluated and monitored on a regular basis. To remove bias and errors from chatbots, modern techniques and a large training set are required^[2,3]^. This is because issues can arise when relying entirely on a large data source. The usage of chatbots raises ethical concerns since it may have unanticipated or unwanted consequences. As AI language models improve, ethical controls and constraints must be implemented to avoid the spread of harmful ideas and inaccurate information.
